# Efficacy and safety of abacavir/lamivudine plus rilpivirine as a first-line regimen in treatment-naïve HIV-1 infected adults

**DOI:** 10.1186/s12981-020-00272-5

**Published:** 2020-05-21

**Authors:** Sharlene Ho, Joshua Guoxian Wong, Oon Tek Ng, Cheng Chuan Lee, Yee Sin Leo, David Chien Boon Lye, Chen Seong Wong

**Affiliations:** 1 Tan Tock Seng Hospital, Singapore, Singapore; 2 Office of Clinical Epidemiology, Analytics and Knowledge, Tan Tock Seng Hospital, Singapore, Singapore; 3 National Centre for Infectious Diseases, Singapore, Singapore; 4 Department of Infectious Diseases, Tan Tock Seng Hospital, Singapore, Singapore; 5 Lee Kong Chian School of Medicine, Nanyang Technological University, Singapore, Singapore; 6 Yong Loo Lin School of Medicine, National University of Singapore, Singapore, Singapore; 7 Saw Swee Hock School of Public Health, National University of Singapore, Singapore, Singapore

**Keywords:** Abacavir, Rilpivirine, Antiretroviral, Treatment-naïve, HIV

## Abstract

**Background:**

The anti-retroviral combination of abacavir/lamivudine plus rilpivirine (ABC/3TC/RPV) is not recommended by international guidelines as the first-line regimen. However, it is potent, well-tolerated, and affordable, especially in resource-limited settings. This study evaluates the efficacy and safety of ABC/3TC/RPV as an initial regimen for treatment-naïve HIV-1 infected patients.

**Methods:**

A retrospective study was conducted in the largest HIV care centre in Singapore, with data collected June 2011 to September 2017. All treatment-naïve HIV-1 infected adults prescribed ABC/3TC as part of their initial anti-retroviral therapy regimen were included. The third drug was a non-nucleoside reverse-transcriptase inhibitor (NNRTI) such as RPV or efavirenz (EFV), or boosted protease-inhibitor (PI). Patients were followed up for 48 weeks. The primary end-point was the percentage of patients achieving virologic suppression, analysed using on-treatment analysis. Secondary outcomes included CD4-count change, treatment discontinuation and treatment-related adverse events.

**Results:**

170 patients were included in the study, 66 patients in the RPV group, 104 patients in the comparator group (EFV or boosted PI). 96% (n = 24) in the RPV group and 87% (n = 26) in the comparator group achieved viral suppression at 48 weeks (p = 0.28). Median (interquartile range) time to viral suppression was similar: 17 (14–24) weeks in the RPV group, and 21 (13–26) weeks in the comparator group. There were no statistically significant differences in the CD4 count between the two groups. 14% (n = 9) of patients on RPV discontinued treatment before 48 weeks, compared to 30% (n = 31) from the comparator group (p = 0.053). Of these, 23 discontinuations were due to drug adverse effects, and only 1 attributed to RPV (p < 0.01). One patient in each group had virologic failure.

**Conclusion:**

RPV is effective, safe and considerably more tolerable than compared to NNRTI or boosted PI in ABC/3TC-containing regimens for treatment-naïve patients. It offers an affordable and attractive option, especially in resource-limited settings.

## Background

Current combination anti-retroviral therapies (cART) against human immunodeficiency virus (HIV) are highly effective. Conventional three-drug cART regimens for treatment naïve patients generally consist of two nucleoside reverse transcriptase inhibitors (NRTIs) and a third drug from another class: integrase strand transfer inhibitors (INSTI), non-nucleoside reverse transcriptase inhibitors (NNRTI) or protease inhibitors (PI). In 2019, the World Health Organisation (WHO) updated its recommendations on the preferred first-line HIV regimen-favouring INSTIs in combination with two NRTIs [1]. Similar recommendations have also been put forth by the United States Department of Health and Human Services (DHHS) and European AIDS Clinical Society (EACS) guidelines [2, 3]. These changes have largely been driven by increasing concerns of the rising HIV drug resistance to NNRTIs, especially in Africa [4]. In Asia, the prevalence of pre-treatment NNRTI resistance is considerably lower, with the yearly increase in the odds of pre-treatment drug resistance reported as 11% (2–20%) [4]. The use of NNRTIs is still recommended as an alternative first-line regimen and in certain settings [1–3]. Besides drug resistance, many factors have to be considered when selecting a cART regimen, such as virologic efficacy, adverse effect profile, comorbid conditions, drug interactions, pill burden, cost and access [2, 3].

NRTI backbone therapies include tenofovir disoproxil fumarate (TDF) or tenofovir alafenamide (TAF) (a prodrug formation of TDF) in combination with emtricitabine (FTC) or lamivudine (3TC), and abacavir and lamivudine (ABC/3TC). ABC/3TC offers the main advantage of avoiding potential renal and bone toxicity seen with tenofovir based regimen and can be used in individuals with renal insufficiency and osteoporosis [5, 6]. It is also safe to be used in the paediatric population [1].

Rilpirivine (RPV) is a second-generation NNRTI approved for use with NRTIs in treatment-naïve HIV-1 patients with pre-treatment viral loads of less than 100,000 copies/ml [2, 3]. Its use is recommended to be limited to patients with pre-treatment CD4 counts exceeding 200 cells/mm^3^ [2, 3]. RPV has fewer reported neurologic and psychiatric adverse effects compared with efavirenz (EFV) [7], and a more favourable metabolic profile compared with protease inhibitors, making it an attractive third drug in combination with NRTIs [8]. The combination of ABC/3TC plus RPV is a once-daily regimen with low pill burden [7]. This combination is also among the most cost-effective regimens because it is relatively inexpensive; and does not require regular monitoring of renal function, urinalysis and bone mineral density as with the use of tenofovir-containing regimens. This is particularly important in settings with limited healthcare resources.

However, data on the use of ABC/3TC in combination with RPV in treatment-naïve patients are scarce. Most patients in the ECHO and THRIVE clinical trials received TDF/FTC as the NRTI backbone, and only 35 patients in the THRIVE study received ABC/3TC plus RPV [9–11]. Current guidelines do not recommend it as an initial regimen for treatment-naïve patients [1–3]. However, in 2016, Curran et al. have reported the effectiveness and safety of ABC/3TC plus RPV in 84 treatment-naïve HIV-1 patients in a retrospective cohort study in Spain [12].

The purpose of this study is to evaluate the efficacy and safety of ABC/3TC plus RPV as an initial regimen for treatment-naïve HIV-1 patients in routine clinical practice in Singapore.

## Methods

### Study design and patients

This was a retrospective study conducted at the National Centre for Infectious Diseases (NCID), which provides HIV care for the largest cohort of HIV-infected individuals in Singapore, between June 2011 and September 2017. All treatment-naïve HIV-1 infected patients (≥ 18 years old) who received ABC/3TC were included. The third drug could either be a NNRTI (RPV or EFV) or boosted PI (atazanavir or darunavir). Exclusion criteria were pre-treatment viral load > 100,000 copies/ml, CD4 count < 200 cells/mm^3^, hepatitis B co-infection (requiring treatment with TDF) and pregnancy.

Data were collected from the NCID HIV Clinical Database, a standardised computerised database containing records of demographic information, HIV transmission route, baseline viral load and CD4 count, AIDS-defining illnesses, cART regimen history and treatment-associated adverse events. Individual patient case records were reviewed to determine underlying medical conditions and to obtain laboratory results such as lipid profile, kidney and liver function tests when these were not available on the Clinical Database. Patients were followed up for 48 weeks from initiation of cART. The interval for follow-up was determined by individual attending physicians as per routine clinical care guidelines. During follow-up, information regarding viral load, CD4 count, laboratory parameters, treatment-related adverse events, and reasons for discontinuing cART, if any, were collected. If cART was stopped due to virologic failure, genotype resistance testing (GRT) was done. Data were anonymised upon extraction. The follow-up was censored for analyses in March 2018.

Virologic response was classified according to the definitions in the DHHS guidelines [2]. Virologic suppression was defined as viral load < 40 copies/ml, based on the lower limit of detection of the laboratory assay used by our institutional laboratory, which determined HIV-RNA levels using the Abbott Realtime HIV-1 assay. Virologic failure was defined as the inability to achieve or maintain viral load < 200 copies/ml. After achieving virologic suppression, a confirmed viral load > 200 copies/ml was considered virologic rebound, whereas an isolated detected HIV-RNA level > 40 copies/ml but < 200 copies/ml that was followed by a return to virologic suppression was considered virologic blip.

The primary efficacy end-point was the percentage of patients who achieved virologic suppression (viral load < 40 copies/ml). This outcome was analysed using on-treatment analysis, in which discontinuation and missing data were censored. The median time taken to achieve virologic suppression was also analysed. Secondary outcomes were changes in CD4 count, treatment discontinuation before 48 weeks, reasons for discontinuation, treatment-related adverse events, and lipid, kidney and liver profile changes.

### Statistical analysis

Univariate analyses were carried out using the Chi square and Fisher’s exact test for categorical variables. Kaplan–Meier survival curves were drawn to show median time to HIV viral load suppression, for the 2 treatment regimes. Log-rank tests were used to test for differences between the 2 survival functions. Cox proportional hazards model was used to obtain the treatment effect sizes. When necessary, follow-up was categorised into 12-weekly intervals. Using Stata MP 13 (Stata Corp, Texas, USA), all analyses were performed at a 5% significance level.

## Results

**Fig. 1:**
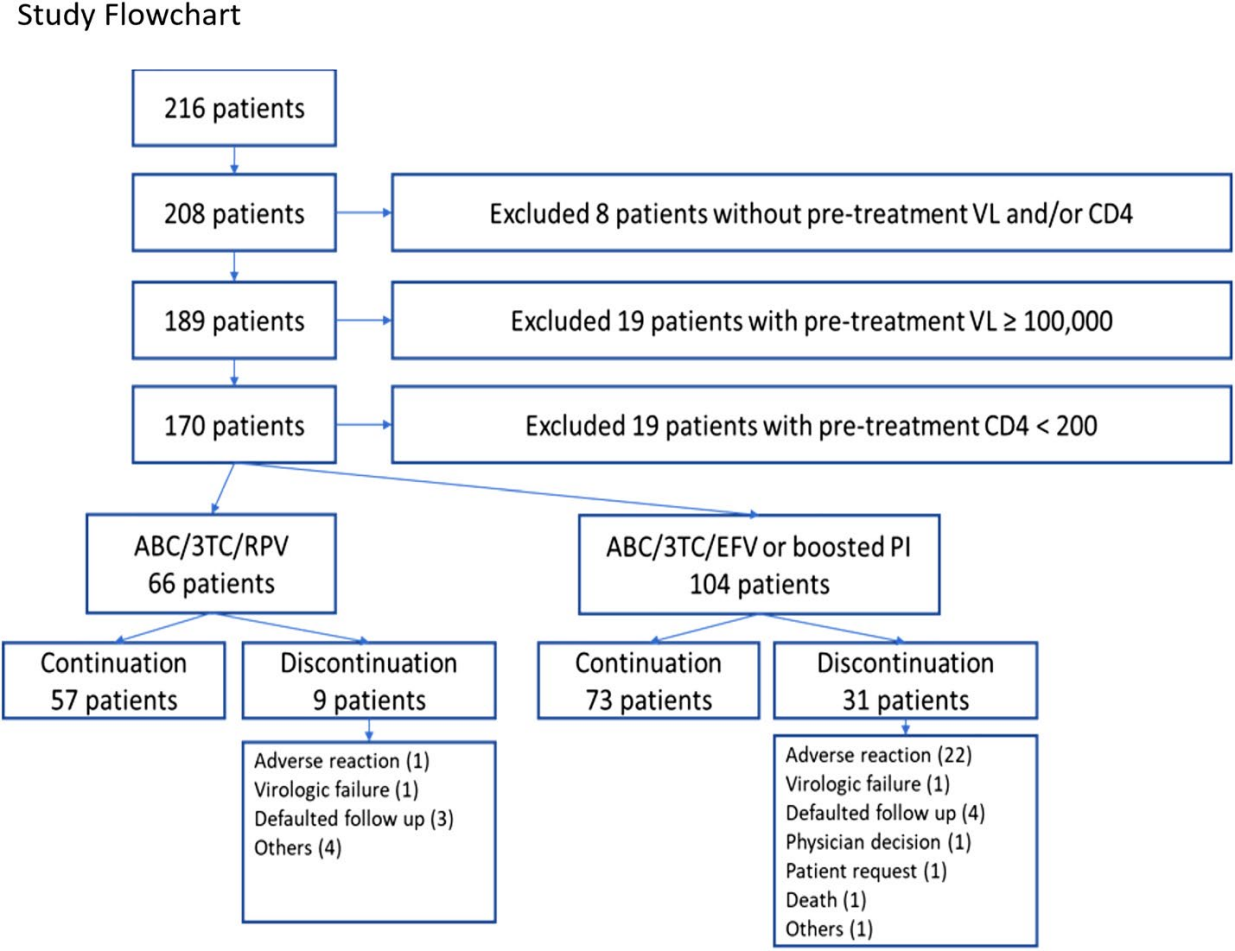
Study flowchart. *ABC* abacavir, *3TC* Lamivudine, *RPV* rilpivirine, *EFV* efavirenz, *PI* protease inhibitor, *VL* viral load

A total of 216 patients were screened, based on the inclusion criteria of being prescribed ABC/3TC as first-line cART. Of these, 170 patients were included in the study after excluding those with incomplete data (n = 8), pre-treatment viral load > 100,000 copies/ml (n = 19) and CD4 count < 200 cells/mm^3^ (n = 19). Sixty-six patients (39%) received ABC/3TC plus RPV and 104 patients (61%) received ABC/3TC plus EFV or a boosted PI (Table 1, Fig. 1, Additional file 1). Of the 104 patients, 89 received EFV, 3 received ritonavir-boosted darunavir and 12 received ritonavir-boosted atazanavir.

**Table 1:** Baseline characteristics of patients

	ABC/3TC/RPV (n = 66) (%)	ABC/3TC/EFV or boosted PIs (n = 104) (%)	p-value
Age, years, median (IQR)	35 (28–46)	30 (30–49)	0.04
Male	63 (95.4)	95 (91.3)	0.37
Race			0.40
Chinese	45 (68.2)	73 (70.2)	
Malay	16 (24.2)	21 (20.2)	
Indian	5 (7.5)	5 (4.8)	
Others	0 (0)	5 (4.8)	
HIV transmission route			0.20
Homosexual	42 (63.6)	54 (52)	
Heterosexual	12 (18.2)	30 (28.9)	
Bisexual	8 (12.1)	18 (17.3)	
IVDU + sexual contact	2 (3.0)	1 (1.1)	
Others	2 (3.0)	1 (1)	
Baseline viral load (copies/ml)			0.42
< 10,000	21 (31.8)	24 (23.1)	
10,000–50,000	34 (51.5)	58 (55.8)	
> 50,000	11 (16.7)	22 (21.2)	
Baseline CD4 count			0.37
200–350	21 (31.8)	44 (42.3)	
> 350–500	21 (31.8)	30 (28.8)	
> 500	24 (36.4)	30 (28.8)	
AIDS defining illness	0 (0)	0 (0)	NA
HCV co-infection	2 (3.0)	3 (2.9)	0.95
Genotype test at diagnosis	29 (44.0)	55 (52.9)	0.26
Time from diagnosis to treatment, days, median (IQR)	67 (45–215)	84 (48–351)	0.27
HLA B*5701			<0.01
Positive	0 (0)	1 (1.0)	
Negative	61 (92.4)	65 (62.5)	
Not done	5 (7.6)	38 (36.5)	
Comorbidities			
Diabetes mellitus	5 (7.5)	6 (5.8)	0.64
Hypertension	7 (10.6)	9 (8.6)	0.67
Hyperlipidemia	5 (7.6)	9 (8.7)	0.80
IHD/CAD	1 (1.5)	2 (1.9)	0.84
Congestive cardiac failure	0 (0)	0 (0)	NA
Chronic liver disease	0 (0)	0 (0)	NA
Chronic kidney disease	0 (0)	3 (2.9)	0.16
Cancer	0 (0)	1 (1)	0.42
CVA	0 (0)	0 (0)	NA
Osteoporosis	0 (0)	0 (0)	NA

*ABC* abacavir, *3TC* lamivudine, *RPV* rilpivirine, *IQR* interquartile range, *IVDU* intravenous drug use, *AIDS* acquired immunodeficiency syndrome, *HCV* hepatitis C Virus, *IHD* ischaemic heart disease, *CAD* coronary artery disease, *CVA* cerebrovascular accident

There were no significant differences in the baseline characteristics between the RPV group and the EFV or boosted PI group (Table 1). Median (Interquatile range, IQR) age at HIV diagnosis was 35 (28–46) years old in the RPV group and 39 (30–49) years old in the EFV or boosted PI group. The majority of patients were males (n = 158, 93%) and Chinese (n = 118, 69%), reflecting the general epidemiology of HIV infection in Singapore. The most common route of HIV transmission was via homosexual transmission (n = 96, 56%). None had AIDS defining illness at diagnosis. Five patients (3%) had HCV co-infection. The median (IQR) time from diagnosis to treatment was 67 (45–215) days for the RPV group, and 84 (48–351) days for the EFV or boosted PI group. No differences in baseline HIV viral load or CD4 count were observed between the groups. Seventy-five percent (n = 127) had Human Leukocyte Antigen (HLA) B*5701 testing, and 49% (n = 84) had HIV GRT done prior to starting treatment. The most common co-morbidities were hypertension (n = 16, 9%), hyperlipidaemia (n = 14, 8%) and diabetes mellitus (n = 11, 6%).

**Fig. 2:**
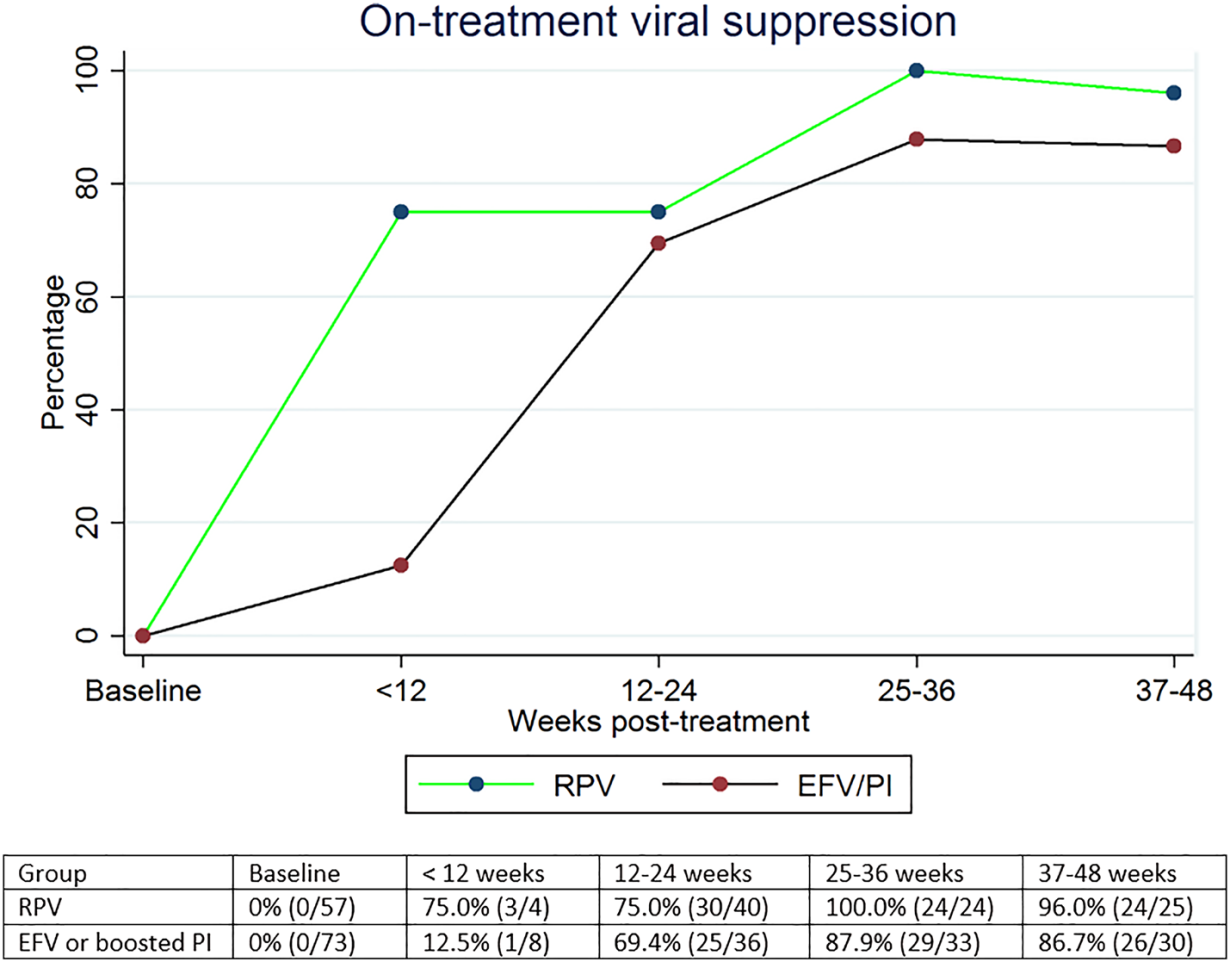
On-treatment viral suppression. *RPV* rilpivirine, *EFV* efavirenz, *PI* protease inhibitor

**Fig. 3:**
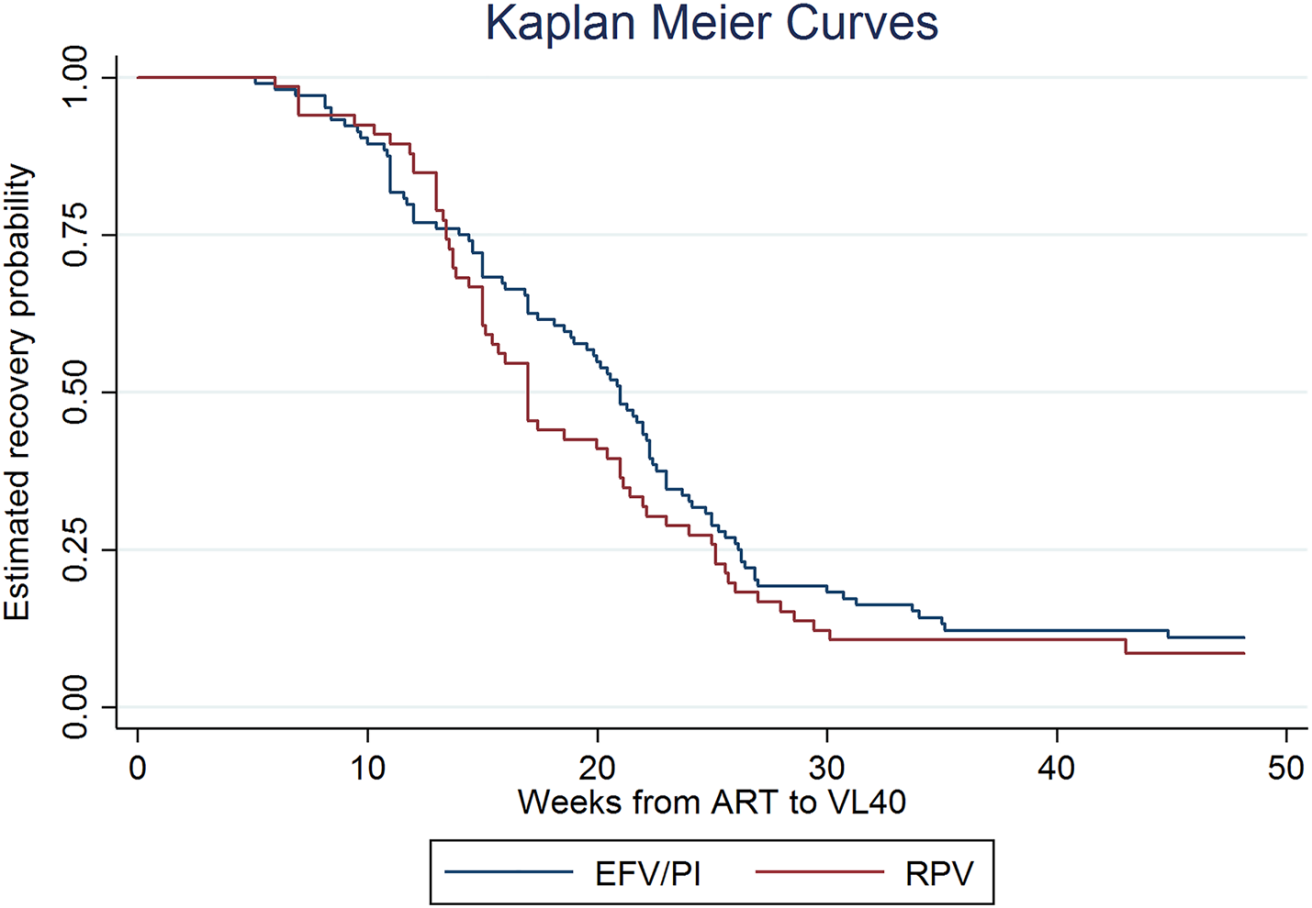
Time taken to achieve virologic suppression. *RPV* rilpivirine, *EFV* efavirenz, *PI* protease inhibitor, *ART* anti-retroviral therapy, *VL40* viral load < 40 copies/ml

**Fig. 4:**
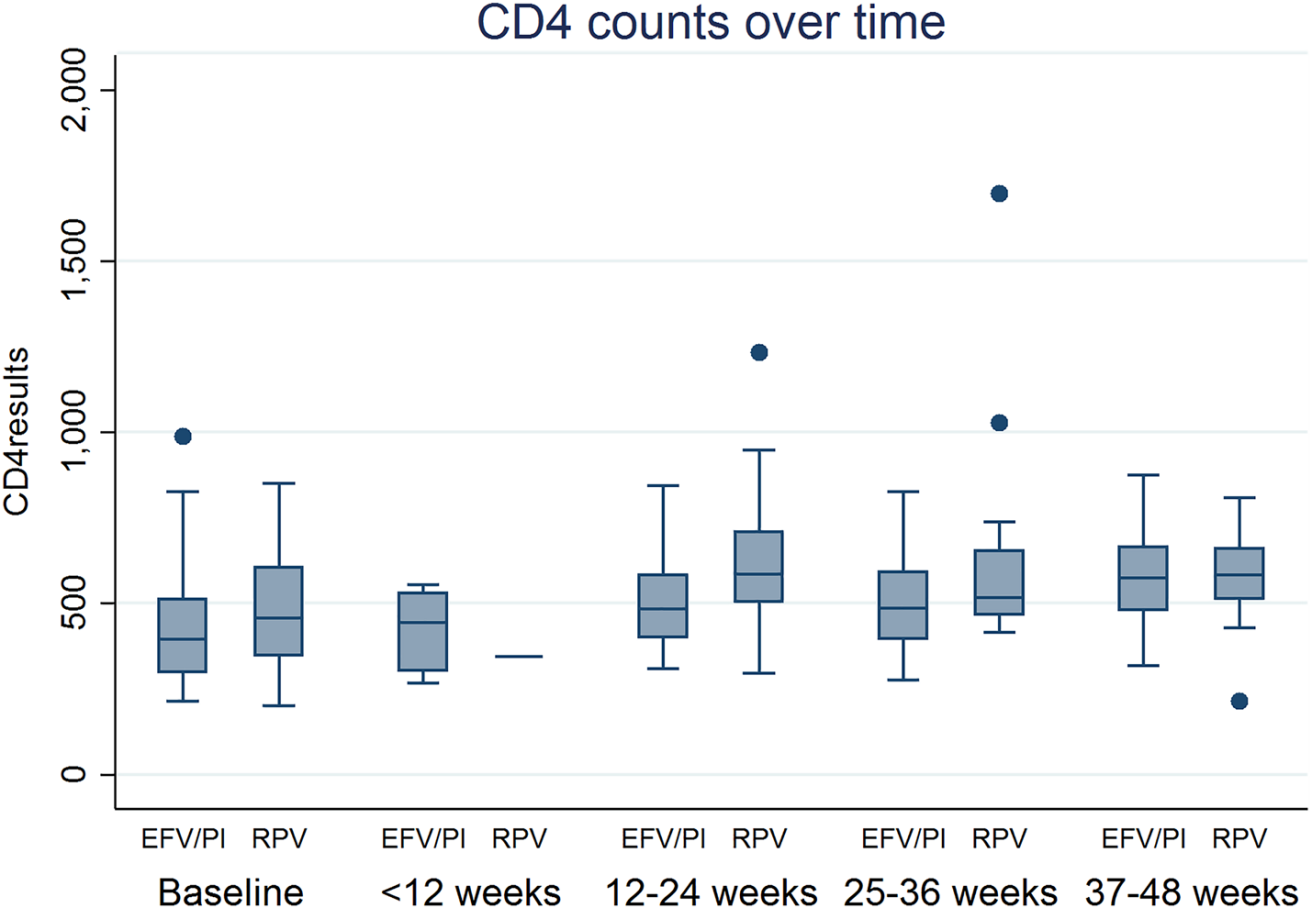
CD4 count over time. *RPV* rilpivirine, *EFV* efavirenz, *PI* protease inhibitor

In the on-treatment analysis, the percentage of patients who achieved virologic suppression at 48 weeks was similar between the two groups. It was 96% (n = 24) in the RPV group and 87% (n = 26) in the EFV or boosted PI group, p = 0.28 (Fig. 2). The median time taken to achieve virologic suppression was also similar (Fig. 3). In the RPV group, median (IQR) time to viral load < 40 copies/ml was 17 (14–24) weeks. In the EFV or boosted PI group, it was 21 (13–26) weeks. There were no statistically significant differences in the CD4 count between the two groups. In the RPV group, the median (IQR) CD4 count changed from 457 (346–604) cells/mm^3^ to 583 (513–660) cells/mm^3^ and in the EFV or boosted PI group, from 394 (297–511) cells/mm^3^ to 574 (479–665) cells/mm^3^ over the 48-week period (Fig. 4).

Nine out of 66 patients (14%) from the RPV group had discontinuation of treatment before 48 weeks, compared with 31 out of 104 patients (30%) from the EFV or boosted PI group (p = 0.05). In the RPV group, 1 patient discontinued treatment due to an adverse reaction, 1 had virologic failure, 3 defaulted clinic follow-up and 4 had other reasons for discontinuation. In the EFV or boosted PI group, the main reason for discontinuation was adverse reactions (n = 22, 71%). One patient had virologic failure, 4 defaulted clinic follow-up, 1 had cART regimen switched based on a decision made by the attending physician, 1 patient requested for cART switch, 1 patient died and 1 had other reasons for discontinuation (Fig. 1) The patient who died was a 53-year-old man with lung cancer, and had received ABC/3TC plus EFV for 36 weeks with virologic suppression before his death.

A total of 23 patients discontinued their initial cART due to adverse reactions (Table 2). Twenty-one had received EFV, 1 had received ritonavir-boosted atazanavir and 1 had received RPV (p < 0.01). The most common adverse reactions were neuropsychiatric side effects (n = 13, 46%) and rash (n = 12, 43%). Most of the adverse reactions were mild and only 3 required treatment or hospitalisation. One patient on ABC/3TC plus EFV tested positive for HLA B*5701. Abacavir was immediately discontinued and switched to tenofovir based regimen when the HLA B*5701 results were available after 1 week on abacavir. He did not experience the abacavir hypersensitivity reaction.

**Table 2:** Adverse reactions resulting in discontinuation of cART, n (%)

Patients with ≥ 1 adverse reaction	23
Total number of adverse reactions	28
Types of adverse reaction	
Dermatological	12 (42.9)
Gastrointestinal/hepatic	2 (7.1)
Neuropsychiatric	13 (46.4)
Endocrine/metabolic	1 (3.6)
Severity of adverse reaction	
Mild (symptoms do not require major medical intervention)	25 (89.3)
Moderate (requires medical treatment or hospitalisation)	3 (10.7)
Severe (fatal or life threatening)	0 (0)

Two patients fulfilled the DHHS guideline criteria for virologic failure. One patient who received ABC/3TC plus RPV had a viral load of 9730 copies/ml at 24 weeks and 26,200 copies/ml at 32 weeks. GRT showed multiple NRTI, NNRTI and PI associated mutations. These included M41L, D67N, M184V, T215Y, K101KE, E138EAG, Y181C, Y188YFHL, and M230ML mutations, many which are associated with high levels of reduced susceptibility to ABC/3TC as well as RPV [13]. He did not have GRT done prior to starting cART. This patient was switched to TDF/FTC plus dolutegravir. Another patient on ABC/3TC plus boosted atazanavir had a viral load of 180 copies/ml at 8 weeks and 133,400 copies/ml at 20 weeks. He was found to be non-adherent to ART because of gastrointestinal side effects, and was later switched to zidovudine/lamivudine plus ritonavir-boosted atazanavir. GRT for this patient revealed pan-susceptible wild-type virus.

Three patients had virologic rebound: 2 from the EFV or boosted PI group and 1 from the RPV group. The first patient had a viral load of 35,136 copies/ml at 48 weeks after achieving virologic suppression previously. He was non-adherent to EFV because of giddiness and drowsiness, and EFV was switched to ritonavir-boosted lopinavir, achieving undetectable viral load thereafter. His GRT showed K103N, reflecting a reduced 20-fold reduced susceptibility to EFV [13]. His GRT done prior to starting cART showed pan-susceptible wild-type virus, indicating treatment emergent resistance. The second patient, also in the EFV group had a viral load of 493 copies/ml at 20 weeks despite previously achieving virologic suppression. His viral load subsequently became suppressed at < 40 copies/ml at 28 weeks without changing cART regimen. The third patient on RPV had a viral load of 220 copies/ml at 36 weeks, which came down to < 40 copies/ml at 52 weeks without changing cART. The second and third patients did not have GRT done.

**Fig. 5:**
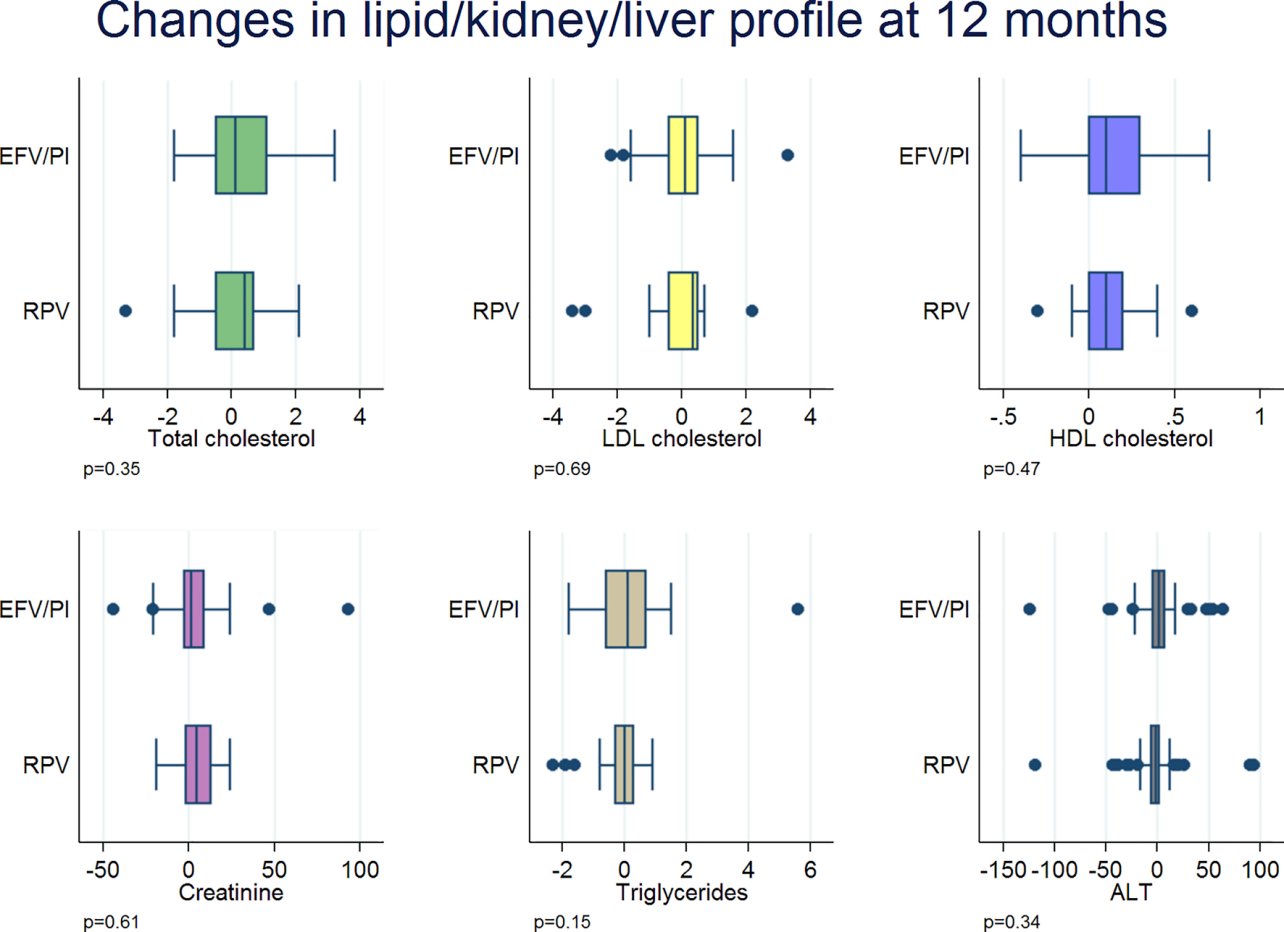
Changes in lipid, kidney and liver profiles at 48 weeks. *RPV* rilpivirine, *EFV* efavirenz, *PI* protease inhibitor, *LDL* low density lipoprotein, *HDL* high density lipoprotein, *ALT* alanine aminotransferase

There were no significant changes in the lipid, kidney and liver profiles at 48 weeks between the two groups (Fig. 5).

## Discussion

Data on ABC/3TC plus RPV as initial regimen in treatment-naïve HIV-1 infected patients are scarce. In the THRIVE study, only 35 patients received ABC/3TC plus RPV, with an overall efficacy of 86%, without apparent differences between different NRTI backbones [10]. In 2016, Curran et al. reported the use of this combination in 84 patients, with effectiveness at 12 months of 97% (on-treatment analysis) and 86% (intention-to-treat analysis). They reported 2 virologic failures and 7 treatment discontinuations (of which one case was due to treatment-related adverse reaction) [12]. Our study showed that ABC/3TC plus RPV can serve as an effective alternative initial regimen for treatment-naïve HIV-1-infected patients with pre-treatment viral loads < 100,000 copies/ml and CD4 count > 200 cells/mm^3^. We report treatment efficacy of 96% at 12 months, which is similar to previous studies in the published literature. In addition, there was no significant difference in the virologic effectiveness compared with the EFV or boosted PI group.

Our study also showed that ABC/3TC plus RPV is a safe and tolerable regimen with fewer adverse reactions and subsequent treatment discontinuations compared with ABC/3TC plus EFV or boosted PI. Most discontinuations were seen in patients receiving ABC/3TC plus EFV. The main reason was neuropsychiatric side effects from EFV such as abnormal dreams, insomnia, headache, giddiness and depression, which are well-described and have prompted the EFV-based cART regimens to be recommended as alternative regimens by the DHHS and other guidelines [2, 14]. RPV, a second generation NNRTI, has fewer neuropsychiatric side effects and is better tolerated, as seen in our study, and evidenced by the fewer discontinuations due to intolerance. This is reflected also in the ECHO, THRIVE and STaR trials utilising the TDF/FTC backbone [9–11, 15]. Although RPV has a better metabolic profile compared with PI, this was not reflected in our study, mainly because there were very few patients on ABC/3TC plus boosted PI, precluding significant differences in metabolic complications to be detected. Kidney and liver function remained stable at 48 weeks, further underlining the safety of ABC/3TC plus RPV.

A significant advantage of using ABC/3TC plus RPV is its once-daily dosing frequency, proffering a regimen with low pill burden and small pill size. It consists of two tablets taken daily, with RPV being one of the smallest ART tablet available at 6.4 mm in diameter [16]. Moreover, the cost of RPV is comparable with that of EFV, and much less than that of PIs in Singapore and many other resource-limited settings. Given that RPV performs similarly to these comparators in terms of efficacy, this lower cost argues for the cost-effectiveness of RPV-containing cART regimens. This has been shown by other investigators in other settings [17]. The combination of simplicity, convenience and affordability of this cART regimen makes it an attractive option for first-line therapy in treatment-naïve patients, especially in settings where cost is an important consideration, such as when co-payment for treatment is required, as is the case in Singapore. These patient-centric factors also increase the likelihood of sustained adherence to the regimen.

Important considerations when using RPV include that it needs to be taken with meals to ensure adequate bioavailability and to avoid co-administration with drugs that reduce gastric acidity such as proton pump inhibitors as RPV absorption is dependent on gastric pH [7, 16]. There is also the potential interaction with drugs that undergo cytochrome P450-mediated metabolism [7, 16]. These issues were not seen in our patients and none required discontinuations because of the above reasons. However, these concerns remain important, especially as comorbidities and polypharmacy become more prevalent in an ageing population of HIV-infected individuals.

Our study has several limitations. It was a retrospective, single centre study with relatively small sample size, and there may have been systematic differences in the two groups that could have served as confounders for the observed differences in outcome. We note a predominance of males in our study population, which reflects the general HIV epidemiology in Singapore, however it may not be representative of other populations. Furthermore, not all patients had the same duration of follow-up or interval between follow-up visits. This reflects variation in clinical practice among clinicians. Despite this, the median time to virologic suppression was approximately 17 weeks for the RPV group and 21 weeks for the EFV or boosted PI group. Longer follow-up of these patients would allow better evaluation of the long-term effectiveness and safety of this regimen. It is also imperative to note the limitations of this regimen: reduced efficacy in patients with high pre-treatment viral load and low CD4 count; the lack of safety data in pregnancy; and potential interactions with commonly used medications such as rifampicin and proton-pump inhibitors.

In conclusion, we present the first analysis that directly compared ABC/3TC plus RPV versus ABC/3TC plus EFV or boosted PI as a first-line regimen in treatment-naïve HIV-1 patients. We found similar efficacy and safety of RPV in combination with ABC/3TC in the clinical setting (Additional file 1).

## Supplementary information

**Additional file 1: Table S1.** Baseline characteristics of patients in the RPV, EFV and boosted PI groups.

## Data Availability

All data generated or analysed during this study are included in this published article.
