# Supplementary material for: Efficacy and safety of abacavir/lamivudine plus rilpivirine as a first-line regimen in treatment-naïve HIV-1 infected adults
Source: AIDS Res Ther. 2020 May 21;17:23. doi: 10.1186/s12981-020-00272-5 (PMC7243331; doi:10.1186/s12981-020-00272-5)
Supplement: Supplementary file 1 — Additional file 1: Table S1. Baseline characteristics of patients in the RPV, EFV and boosted PI groups. [file 12981_2020_272_MOESM1_ESM.docx]

**Additional file Table S1. Baseline characteristics of patients in the RPV, EFV and boosted PI groups**

|  | ABC/3TC/RPV (n=66) (%) | ABC/3TC/EFV  (n=89) (%) | ABC/3TC/boosted PIs (n=15) (%) |
| --- | --- | --- | --- |
| Age, years, median (IQR) | 35 (28-46) | 39 (30-49) | 35 (28-47) |
| Male | 63 (95.4) | 81 (91.0) | 14 (93.3) |
| Race  Chinese  Malay  Indian  Others | 45 (68.2)  16 (24.2)  5 (7.5)  0 (0) | 62 (69.7)  18 (20.2)  5 (5.6)  4 (4.5) | 11 (73.3)  3 (20.0)  0 (0)  1 (6.7) |
| HIV transmission route  Homosexual  Heterosexual  Bisexual  IVDU + Sexual contact  Others | 42 (63.6)  12 (18.2)  8 (12.1)  2 (3.0)  2 (3.0) | 45 (50.6)  26 (29.2)  16 (18.0)  1 (1.1)  1 (1.1) | 9 (60.0)  4 (26.7)  2 (13.3)  0 (0)  0 (0) |
| Baseline viral load (copies/ml)  <10,000  10,000 – 50,000  >50,000 | 21 (31.8)  34 (51.5)  11 (16.7) | 18 (20.2)  54 (60.7)  17 (19.1) | 6 (40.0)  4 (26.7)  5 (33.3) |
| Baseline CD4 count  200-350  >350-500  >500 | 21 (31.8)  21 (31.8)  24 (36.4) | 38 (42.7)  26 (29.2)  25 (28.1) | 6 (40.0)  4 (26.7)  5 (33.3) |
| AIDS defining illness | 0 (0) | 0 (0) | 0 (0) |
| HCV co-infection | 2 (3.0) | 3 (3.4) | 0 (0) |
| Genotype test at diagnosis | 29 (44.0) | 46 (51.7) | 9 (60.0) |
| Time from diagnosis to treatment, days, median (IQR) | 67 (45-215) | 48 (85-448) | 33 (73-206) |
| HLA B*5701  Positive  Negative  Not done | 0 (0)  61 (92.4)  5 (7.6) | 1 (1.1)  52 (58.4)  36 (40.4) | 0 (0)  13 (86.7)  2 (13.3) |
| Comorbidities  Diabetes mellitus  Hypertension  Hyperlipidemia  IHD/CAD  Congestive cardiac failure  Chronic liver disease  Chronic kidney disease  Cancer  CVA  Osteoporosis | 5 (7.5)  7 (10.6)  5 (7.6)  1 (1.5)  0 (0)  0 (0)  0 (0)  0 (0)  0 (0)  0 (0) | 5 (5.6)  9 (10.1)  9 (10.1)  2 (2.2)  0 (0)  0 (0)  2 (2.2)  1 (1.1)  0 (0)  0 (0) | 1 (6.7)  0 (0)  0 (0)  0 (0)  0 (0)  0 (0)  1 (6.7)  0 (0)  0 (0)  0 (0) |

ABC: Abacavir; 3TC: Lamivudine; RPV: Rilpivirine; IQR: Interquartile Range; IVDU: Intravenous Drug Use; AIDS: Acquired Immunodeficiency Syndrome; HCV: Hepatitis C Virus; IHD: Ischaemic Heart Disease; CAD: Coronary Artery Disease; CVA: Cerebrovascular Accident
